# Points-to-consider on the return of results in epigenetic research

**DOI:** 10.1186/s13073-019-0646-6

**Published:** 2019-05-23

**Authors:** Stephanie O. M. Dyke, Katie M. Saulnier, Charles Dupras, Amy P. Webster, Karen Maschke, Mark Rothstein, Reiner Siebert, Jörn Walter, Stephan Beck, Tomi Pastinen, Yann Joly

**Affiliations:** 1 Centre of Genomics and Policy, Faculty of Medicine, McGill University, Montreal, Quebec H3A 0G1 Canada; 2 Montreal Neurological Institute, Faculty of Medicine, McGill University, Montreal, Quebec H3A 2B4 Canada; 3 UCL Cancer Institute, University College London, London, WC1E 6DD UK; 4 The Hastings Center, Garrison, NY 10524-4125 USA; 5 Institute for Bioethics, Health Policy and Law, University of Louisville School of Medicine, Louisville, KY 40202 USA; 6 Institute of Human Genetics, Ulm University Medical Center, 89091 Ulm, Germany; 7 Saarland University, 66123 Saarbrücken, Germany; 8 Department of Human Genetics, McGill University and Genome Quebec Innovation Centre, Montreal, Quebec H3A 0G1 Canada; 9 Center for Pediatric Genomic Medicine, Children’s Mercy Kansas City, Kansas City, MO 64108 USA

**Keywords:** Return of results, Incidental findings, Epigenetics, ELSI

## Abstract

As epigenetic studies become more common and lead to new insights into health and disease, the return of individual epigenetic results to research participants, in particular in large-scale epigenomic studies, will be of growing importance. Members of the International Human Epigenome Consortium (IHEC) Bioethics Workgroup considered the potential ethical, legal, and social issues (ELSI) involved in returning epigenetic research results and incidental findings in order to produce a set of ‘Points-to-consider’ (P-t-C) for the epigenetics research community. These P-t-C draw on existing guidance on the return of genetic research results, while also integrating the IHEC Bioethics Workgroup’s ELSI research on and discussion of the issues associated with epigenetic data as well as the experience of a return of results pilot study by the Personal Genome Project UK (PGP-UK). Major challenges include how to determine the clinical validity and actionability of epigenetic results, and considerations related to environmental exposures and epigenetic marks, including circumstances warranting the sharing of results with family members and third parties. Interdisciplinary collaboration and good public communication regarding epigenetic risk will be important to advance the return of results framework for epigenetic science.

## Background

Epigenetics is a fast-growing field of research that is shedding light on the ways in which interactions with the environment lead to changes in gene expression [1]. Over the past 20 years, some of the most concerning diseases of our time, such as many types of cancer, metabolic disorders, and neurodegenerative diseases, have been associated with the disruption of epigenetic programs [2–6]. Processes such as aging and personal exposure to stress and trauma have also been associated with altered epigenetic programs [7].

As human epigenome mapping and epigenetic research continue to progress, with the potential to influence our understanding of environmental exposures, community health, and the health of future generations [8–10], determining which individual epigenetic research results might be communicated to research participants and how this communication should take place are of growing importance. The return of research results and incidental findings is a topic that has been explored at great length, mostly in the fields of genetics and imaging [11–14]. Although epigenetic research is still in its infancy, it is expected to elucidate many aspects of human health. Scientific and bioethics considerations already point to a number of areas where the potential risks and challenges of the return of research results might differ in type or scale from those relating to genetic data [15–19], and researchers have called for further guidance on the subject [17, 19]. These differences are likely to impact notions of the clinical validity and actionability of epigenetic results, privacy considerations, and assessment of the circumstances that warrant the sharing of results, both with the research participants themselves and with other individuals who may be concerned (for example, those who have had similar environmental exposures).

The International Human Epigenome Consortium (IHEC) is an international consortium with the objective of ‘providing free access to high-resolution reference human epigenome maps for normal and disease cell types to the research community’ [20, 21]. The IHEC Bioethics Workgroup, an interdisciplinary group of researchers in science, ethics, policy, and the law, therefore formed a Subgroup to anticipate and consider the ethical, legal, and social issues (ELSI) raised by the return of epigenetic research results. This Subgroup has produced a set of points-to-consider (P-t-C) for the community, which has been approved by the Bioethics Workgroup and IHEC Executive Committee.

## Points-to-consider

Building on the consensus that has emerged from the genetics literature and international ethics guidance [22, 23], we recognized that: “The view is becoming more common [ …] that clinically valid and actionable individual research results should be offered to participants” (Box 1, P-t-C point 1). This is not meant to imply that further results should not be returned under certain circumstances, but clinically valid and actionable individual results, whether they are incidental findings or directly related to the research study, represent a minimum threshold for the type of results to be considered. Our P-t-C also stress, however, that “researchers are not expected to actively search for this information (all clinically valid and actionable individual results) unless it forms part of their standard research practice” (Box 1, P-t-C point 1), as doing so would create an undue burden on researchers. Furthermore, the definitions of the terms ‘clinically valid’ and ‘actionable’ are not yet as well-established in the epigenetics field as they are in genetics. Therefore, we identified a number of characteristics and considerations concerning epigenetic data that could help researchers to determine which results should be returned according to the two criteria of clinical validity and actionability.

Other, more procedural recommendations were derived and adapted from guidelines and literature on the return of genetic results. These included the well-established requirement that results be returned only when the participant has accepted to receive the results after having been given the option of agreeing or declining to this through an informed consent process [22, 24].

We also warn of the possibility that epigenetic information may not be protected under genetic non-discrimination laws because these laws use language that is specific to genetics and may not cover all epigenetic data. For example, such laws refer to genetic characteristics that are acquired before birth (in Germany [25]) or to ‘DNA’, ‘RNA’, or ‘genotypes’ (in the United States [26] and Canada [27]) [28–31]. Given the uncertainty about whether genetic non-discrimination laws apply to epigenetic data, some individuals may be reluctant to enroll in specific epigenetic studies or to give broad consent to the use of their biospecimens in research that could result in analysis of their epigenetic information. Thus, genetic non-discrimination laws may need to be applied in a way that includes epigenetic data, or new laws focusing specifically on epigenetics may need to be enacted.

Finally, ethical issues related to the disclosure of incidental findings or the return of results will depend on the age and cognitive capacity of the research participant, including the potential for prenatal epigenetic testing. For example, it may be preferable to offer certain results, such as the risk of adult-onset conditions, to children once they are able to consent to this themselves [32]. Furthermore, it may not be appropriate for parents or legally authorized representatives to refuse to receive actionable results on behalf of children or incapable adults [33]. Although this point is not specific to epigenetics, we adopt a point on the need to develop specific policies for the contexts of research in pediatrics and research involving adults who have been deemed incapable of giving informed consent (Box 1, P-t-C point 8). This need is well-established in guidelines for the return of genetic results [14, 34, 35].

Having grounded our P-t-C in current guidance in the field of genetics, our overarching aim was to bring attention to the particular issues associated with epigenetic research data: the challenges that lie ahead for determining clinical validity and actionability in epigenetics; considerations related to environmental exposures and epigenetic marks, including their impact on the sharing of results with others; and, finally, the importance of good communication regarding epigenetic risk (Box 1, P-t-C points 2–5).

### Clinical validity of epigenetic research results

Definitive molecular diagnosis of imprinting disorders, such as Beckwith–Wiedemann syndrome (which is mainly caused by genetic or epigenetic defects in the chromosome 11p15.5 region), can sometimes be reached by analysis of epigenetic marks alone [36]. Even for this very rare group of diseases, however, an underlying DNA sequence change (mutation) is commonly required to return a clinical diagnosis. Given the current uncertainty regarding the clinical significance and application of the vast majority of epigenetic data, returning clinically valid, actionable results from epigenetic research studies would require a careful process of scientific and clinical review, both across the field and of individual study results. As more systematic evidence of the epigenetic causes of disease is only beginning to emerge from large-scale epigenome projects [21, 37–41], the establishment of exhaustive criteria for assessing the clinical validity and actionability of epigenetic data would be premature at this time. Therefore, we focused on framing in general terms how epigenetic evidence might eventually compare to genetic data, drawing on the criteria and scoring systems that have evolved over many years to assess the significance and clinical interpretation of genetic variants [42–46]. This entailed breaking down the assessment of epigenetic data that could potentially be communicated to participants into the following constituent areas:

1. The accuracy of the epigenetic data with respect to both the technology used and the source material (cell composition, sample purity).
2. The stability of the epigenetic data. Some epigenetic marks are more dynamic than others, so multiple measurements over time might be required to determine their significance [47, 48].
3. The existing level of evidence that a variant or mark may cause disease or is associated with disease, the magnitude of such disease risk, and the nature of the disease.
4. And finally, the possibility of treating or preventing disease or epigenetic risk variants (for example, by systemic or targeted epigenetic therapy, or through epigenetic screening).

In addition, we proposed specific terminology to conceptualize the typical levels of evidence that are found in discussions of epigenetic risk and disease. Disease-associated or disease-causing variants would thus fall into one of the following groups:

1. Associated variants: variants supported by statistics only (for example, in an epigenome-wide association study (EWAS)).
2. Inferred variants: variants supported by statistics and inferred functional evidence (for example, involvement in a plausible mechanism that has been inferred from additional data).
3. Causal variants: variants supported by statistics and for which disease-causality has been demonstrated (for example, in conjunction with genetic variants or where genetic variants have been ruled out). Causal variants are candidates for clinical validation as a first step towards actionability.

We also point out that epigenetic variants or marks may be diagnostic or useful as ‘biomarkers’ of disease, even if they are not causal (Box 1, P-t-C point 2e). They may also be found to confer protection against disease. We hope that these categories will serve as a starting point for defining levels of evidence in different areas of epigenetics, as has been done in evaluating the clinical validity of gene–disease associations, for example, by the Clinical Genome Resource (ClinGen) [46, 49]. ClinGen is an initiative to provide an authoritative central resource that defines the clinical relevance of genes and genetic variants for use in precision medicine and research. Approaches that are commonly used to demonstrate the causality of epigenetic variants are genetic manipulation of the DNA sequence underlying an epigenetic variant or of the enzymes that are responsible for the establishment or removal of the epigenetic variant, or targeted editing of the epigenetic variant itself [50].

Although we acknowledge that epigenetic variants and their clinical interpretation may differ considerably from genetic variants, we aimed to achieve two goals with this preliminary framework. First, to place the epigenetic research result that a researcher may be considering communicating in the context of a thorough assessment of its analytical, scientific and clinical validity. Second, to frame the result in terms of its likely impact on participants, both in its relevance to participant health and its broader significance. This is particularly complex because of evidence that epigenetic marks may be reversible [48] and may sometimes provide information about an individual’s environmental exposures [51], including information that might be related to their and to others’ behavior [52].

### Actionability of epigenetic research results

We considered that ‘actionability’—the potential for action based on the epigenetic data that are returned—should extend beyond strict definitions of clinical utility to include health-related data more broadly. For example, epigenetic data might indicate an environmental or community exposure, resulting in epigenetic risk variants that could be avoided, such as acceleration of the accumulation of altered DNA methylation biomarkers of aging (the epigenetic clock) [53]. Actionability could therefore include clinical actions to prevent or treat disease or epigenetic risk variants, as well as non-clinical actions that could be enabled by knowledge of the epigenetic data, such as health-related life choices, including reproductive decisions (for example, changing diet or other behaviors that might be involved in health-related epigenetic variation).

The scope of the data that may potentially be of interest to participants is wide, and we certainly did not intend to suggest returning all results in all circumstances. In particular, we include a point about considering the magnitude and nature of the disease risk in weighing the significance of a result (Box 1, P-t-C point 2d). Current policies for the return of genetic information suggest that the ‘severity’ of the disease to which an individual would be predisposed is likely to be important in deciding how critical the return of a result may be [54]. Epigenetic reversibility may also strengthen the ethical argument in favor of disclosing an epigenetic research result, as it may allow for greater preventive or treatment opportunities. On the other hand, it may also lead to data that are not a definitive indication of an individual’s epigenetic disease risk—hence our specific point about the stability of epigenetic data (Box 1, P-t-C point 2b).

Examples of behaviors and other so-called ‘lifestyle’ exposures with known epigenetic effects include nutrition, smoking, and stress [55–58]. While their inference is not yet unequivocal, at least quantitatively, especially for the more intangible exposures such as exposure to stress, it is possible that such individual research results could be of interest to research participants. Research in this area, and into other environmental exposures, is growing [59, 60]. For example, the National Institute of Environmental Health Sciences (NIEHS) Toxicant Exposures and Responses by Genomic and Epigenomic Regulators of Transcription (TaRGET) II Consortium recently reported its plans to investigate the conservation of environmentally induced epigenetic alterations across tissues following environmental exposures that have been associated with adverse health outcomes [61]. As exposure science has moved from measuring chemicals in the environment to biomonitoring of such exposures in the population, novel models of community-driven return of results and broader communication plans are emerging [62].

Individual epigenetic information may be of interest to participants who simply wish to know about their own health status or to influence community health decisions. However, such information also has potential implications that extend to the area of public policy, and more specifically, to areas of environmental tort (where injury occurs via toxic exposure) and reproductive tort (where injury occurs either pre-conception or in utero) [63, 64]. For example, evidence is emerging that toxicity from exposure to certain chemical hazards is driven at least in part by epigenetic mechanisms, and researchers have expressed concerns that assisted reproductive technologies may cause epigenetic damage to embryos [28, 64, 65]. Both environmental and reproductive torts are founded on responsibility for harmful exposure and involve proof of three elements: breach of duty, causation, and injury [64, 66, 67]. Of these, the causal element presents a particular conundrum in environmental and reproductive tort because the scientific evidence is not always sufficiently clear to establish a direct causal link between the action entailed in the breach of duty and the harm suffered by the plaintiff [66, 68]. Although evidence of general causation is often provided by epidemiological data, evidence of specific causation requires a more fine-grained understanding (most of the time not available) of the biological mechanisms underlying such statistical associations between exposure and harm. By providing insights at the molecular level into how significant health risks may be acquired through different manners of exposure, epigenetic research could fill the existing gap in establishing actionable evidence of specific causality [67, 69].

Finally, a few studies of transgenerational epigenetic effects, mainly in mouse models, indicate that environmental and behavioral epigenetic signatures could be inherited [8–10, 52, 70–73]. This possibility, if confirmed, might add to the range of research data that could potentially be of interest to individuals, but it may also raise particular privacy concerns because the data would not only expose the environmental and behavioral information of the research participant, but also possibly that of their parents and grandparents.

### Disclosure of participants’ epigenetic data to relatives and third parties

Disclosing personal genetic information to biological relatives can sometimes benefit family members who share similar genetic-risk profiles. Even though some patients may be reluctant to disclose—for instance, to prevent unnecessary anxiety for family members [74]—some ethicists have argued that there may sometimes be a moral ‘genetic responsibility’ to share medically relevant information with interested third parties [75, 76]. Nevertheless, this moral responsibility can conflict with the obligation of physicians and researchers to protect patient confidentiality, and physicians may also be legally required to inform a patient about the potential ‘consequences that his or her silence may have on the health of family members’ [77]. In the United States, federal health privacy regulations prohibit the nonconsensual disclosure of health information except in circumstances inapplicable here, such as disclosures to public health or law enforcement officials [78]. The superior approach is for health care providers to counsel, encourage, and support patients to disclose relevant genetic information to their at-risk relatives [79].

Ethical and legal debates about the disclosure of genetic information to third parties have consistently focused on the relevance of such information to the patient’s immediate family, that is, to ‘biological relatives’ who are likely to share innate risks acquired through Mendelian inheritance [80]. However, epigenetic research may soon force us to expand the notion of biological relative—and thus the range of people who could benefit from the disclosure of epigenetic information—to include ‘individuals with shared exposures’, that is, individuals who are likely to share similar epigenetic risk factors [81]. This approach will also benefit researchers who investigate DNA sequence mutations and other changes that are induced by environmental exposures [82].

Consider the following hypothetical scenario: numerous studies have shown that a pesticide causes specific epigenetic changes and phenotypes at the population level. An investigator finds out that one of the research participants in their study, who has worked as a farmer all their life, has these epigenetic marks of exposure to the pesticide. Therefore, the exposure occurred in all likelihood at the workplace. Such a research result could carry a number of direct clinical implications for different ‘categories’ of individuals other than the participant, such as: 1) the farmer’s family (including non-genetically related family members such as adopted children) living near the contaminated site, as well as neighbors, as this information could influence decisions surrounding re-location away from the harmful exposure; 2) employees on the farm who may also be at risk of epigenetic effects resulting from frequent exposure to the pesticide; and 3) these individuals’ future children, if there were a risk of possible transgenerational effects.

### Example of returning epigenetic results: Personal Genome Project UK

The Personal Genome Project UK (PGP-UK) [83] conducted a small pilot trial in 2016 to gain experience and a first insight into any issues associated with reporting incidental epigenetic findings to study participants. Using open consent and open access data sharing protocols [84], PGP-UK recruited ten volunteers who agreed to receive incidental epigenetic findings from the analysis of their DNA methylomes in addition to their standard genome reports. Three categories of findings were reported (sex, age, and smoking), for which the analysis was judged to be sufficiently mature based on independent validation and replication. The methylome reports [85] were based on the analysis of around 450,000 genome-wide CpG sites in two specimens (blood and saliva) from each participant [86].

In this small initial trial, there was high participant interest in, and acceptance of, receiving incidental epigenetic findings, as assessed through discussion groups and follow-up with volunteers, particularly the results associated with environmental exposures [86]. This supports our view that results other than clinically actionable results are potentially of great interest to research participants. It also provides limited evidence that participants could also be comfortable with receiving results of uncertain clinical significance, although the level of support provided for the return of results communication process in this trial may not be as feasible for studies involving much larger groups of research participants. Although we expect good communication practices to improve participant understanding of individual results and encourage such efforts, we would not assume that personal preferences regarding the receipt of results would necessarily differ in the absence of such support. Indeed, social science studies have shown that the vast majority of participants in genetics research and biobanking initiatives wish to receive individual results [87–95]. Furthermore, a large multi-study survey found that providing a choice of different consent and data sharing models did not have a significant impact on willingness to participate in a biobank [96].

## Conclusions and future directions

With these P-t-C, we aimed to draw attention to the ELSI associated with the return of epigenetic research results and we have outlined both the norms that have emerged for genetic research results that are relevant and new issues to consider for epigenetic research.

Much remains to be determined before we can arrive at detailed guidance for the return of specific epigenetic results, such as the recommendations that have been produced for clinical genome sequencing in the USA [97, 98]. This will involve considerable research efforts to better understand fundamental epigenetic and epigenomic processes and their relationship to disease, as well as studies of the clinical validity and actionability of epigenetic data. We believe, however, that discussions about the strength of epigenetic findings and their implications for health and disease must begin now, while our understanding of the role of epigenetics is growing. Although we found it useful to build on ELSI guidance from the field of genetics, epigenetic data raise important new challenges that may eventually lead to a very different framework for the return of results.

Furthermore, as epigenetics is attracting much scientific interest and investment, its health implications and potential to revolutionize the ‘nature versus nurture’ debate have also caught the imagination of the public [99–101]. We focused here on the return of individual research results to participants, but the issues of the broader communication and public understanding of epigenetics should not be left out of the discussion. These issues will, in all likelihood, frame both participants’ eventual understanding of any individual research results and the broader societal debate on the implications of epigenetic science. Enhanced approaches for communication with research participants, such as the development of online ‘research portals’ to access and discuss research findings, could provide the public with greater opportunities for interaction with research studies and their results. With these P-t-C, we hope to stimulate innovative, interdisciplinary public conversations about epigenetics and the implications of this science for individuals, families, and societies.

Box 1 IHEC Points-to-consider on the return of epigenetic research results^*^

1. The view is becoming more common in the scientific, bioethics, and policy literature and in ethical guidelines that clinically valid and actionable individual research results should be offered to participants. However, it is agreed that researchers are not expected to actively search for this information (all clinically valid and actionable individual results) unless it forms part of their standard research practice.
2. In determining the clinical validity and actionability of epigenetic data and communicating epigenetic risk, the following points should be considered:
  - How accurate are the data? Consider the study’s quality-control processes and replication of measurements in a clinically accredited diagnostic laboratory before returning research results. Also consider the origin or source of the epigenetic data, which may be important for its interpretation, that is, the cell and tissue composition, and the age and sex (not gender) of the individual.
  - Epigenetic marks may be dynamic; how stable are the acquired data (are they ‘temporarily stable’)? The research result might require multiple samples at different time points to determine its stability.
  - Epigenetic variants or marks have the potential to cause disease. Depending on supporting evidence, three types of variants can be distinguished:
    Associated variants: variants supported by statistics only (for example, in an epigenome-wide association study).
    Inferred variants: variants supported by statistics and inferred functional evidence (for example, involvement in a plausible mechanism that has been inferred from additional data).
    Causal variants: variants supported by statistics and for which disease-causality has been demonstrated (for example, in conjunction with genetic variants or where genetic variants have been ruled out). Causal variants are candidates for clinical validation as a first step towards actionability.
  - For clinically valid variants, what is the level of disease risk and severity?
  - Epigenetic variants or marks may be diagnostic or a ‘biomarker’ even if they are not causal.
  - The possibility of treatment or prevention based on the research result, including the potential ‘reversibility’ of epigenetic risk variants. ‘Actionability’ may also include the possibility of making life choices on the basis of the result.
3. Research results may include epigenetic marks from different kinds of exposures (for example, pollution or certain behaviors) that fall short of disease-causality, yet which are of interest to participants (for example, enabling them to avoid further potentially harmful exposures).
4. As epigenetic data result from both heredity and environmental exposures, individuals who might benefit from receiving this information through further disclosure could eventually include research participants’ non-biological relatives, neighbors, co-workers, or others with shared exposures. Such disclosure should only be made with the participants’ and other individuals’ consent or in accordance with local laws and policies.
5. Public communication of the general results of epigenetic research may have an important, yet often neglected, impact on how individuals interpret their individual epigenetic results. Few epigenomic research projects currently produce clinically valid and actionable individual research results, but many are generating research findings that are of interest to the public and to the media. Good public communication of epigenetic risk by researchers and science communication professionals should be encouraged.

Procedural points

6. An epigenomic project should have a policy on return of research results in place, which is included in the ethics review for the project, and is clearly explained to participants during the informed consent process prior to any sample collection. The policy should include transparency about how results will be assessed for potential return of results. For fundamental research projects that are not meant to generate clinically valid, actionable results, there should be a statement that results will not be returned, except in the exceptional circumstance where unforeseen findings arise that are clinically valid and actionable, and recontact and consent of participants is feasible (for example, if data are not irreversibly de-identified).
7. The return of research results should occur with the free and informed consent of adult participants, in a way that respects their autonomy, including their right to decline the information if they so choose (the ‘right not to know’).
8. Specific policies should be established for pediatric research and for research involving adults who have been deemed incapable of giving informed consent. For example, it may not be appropriate for parents or legally authorized representatives to refuse to receive actionable results on behalf of children or incapable adults.
9. Elements to consider in setting up procedures for offering the return of results include:
  - the expiration of any duty to return results (for example, at the end of the research project);
  - the estimated cost of the process;
  - human resources that will be involved (for example, genetic counselors, family physicians, and others) and the respective roles of researchers and physicians;
  - the necessity of establishing a convenient procedure to collect and update the contact details of participants and to re-identify them if warranted;
  - the potential privacy and security risks of holding participant identities and contact information and ways to mitigate these risks;
  - the approach that will be taken regarding the disclosure of results to family and other potentially exposed individuals depending on laws and jurisdictions;
  - the possibility that epigenetic information may not be protected information under genetic information anti-discrimination laws in a given jurisdiction, and the need to adapt procedures accordingly. Participants should be aware of any additional risks that this issue presents at the time of the initial consent to sample collection.

^*^ Also available from the IHEC website [102]

## Abbreviations

ELSI: Ethical, legal, and social issues
IHEC: International Human Epigenome Consortium
PGP-UK: Personal Genome Project UK
P-t-C: Points-to-consider
